# Supplementary material for: Elucidating the effect of drug-induced mitochondrial dysfunction on insulin signaling and glucose handling in skeletal muscle cell line (C2C12) in vitro
Source: PLoS One. 2024 Sep 17;19(9):e0310406. doi: 10.1371/journal.pone.0310406 (PMC11407670; doi:10.1371/journal.pone.0310406)
Supplement: Attachment — Submitted filename: Plos One Rebuttal letter.pdf [file pone.0310406.s002.pdf]

RE: Elucidating the effect of drug-induced mitochondrial dysfunction on insulin signaling and glucose handling in skeletal muscle cell line (C2C12) *in vitro*

Dear Editor and reviewers

We would like to take this opportunity to convey our gratitude to reviewers for taking their time in reviewing our work and provide insightful comments. We believe such significant inputs have extensively improved the quality of work presented. We have tried to revise the manuscript as per the reviewers' inputs at our best capabilities

Kindly find our responses for each point raised below

Yours sincerely

Dr N Sibiya

Reviewer #1: GENERAL COMMENTS
The study tested the effects of several compounds to alter mitochondrial function and induce insulin resistance. There were some English/grammar issues that a thorough re-read can fix. Although the approaches were appropriate and the data appeared to be carefully obtained, the significant issue was that the drug studies were not completed on insulin treated myotubes.

We thank the reviewer for the comments.

We have substantively revised the manuscript for English and Grammatical issues.

. Our goal aimed to understand how exposure of cells to mitotoxic agents could lessen insulin' effects. Hence our approach aimed to pre-treat the cells with these mitotoxic drugs, followed by insulin treatment. In this way, we could be able to observe changes in insulin sensitivity post-exposure to mitotoxic agents such as glucose uptake and GLUT4 translocation. We believe through this approach, we have been able in part to prove that drug-induced mitochondrial dysfunction (using 6 separate classes of drugs) does affect insulin sensitivity as evidenced by a decrease in insulin-stimulated glucose uptake, AKT, and GLUT 4 translocation. We believe the experiment proposed by a reviewer could be a significant stand-alone study, as it is a different approach, considering that the cells would be pre-treated with insulin following by mitotoxic agents. As such, preliminary experiments will also be vital to establish time-points where one can observe the effects. We welcome the reviewer's comment, however from what we have outline above together with logistical issues associated studies of this nature, we believe this can be conducted as a follow-up study. We would like to believe the current observation broadly shed light on the effect of mitochondrial dysfunction on insulin effect.

SPECIFIC COMMENTS
Page 17, Results, line 6-7. The sentence "However, efavirenz, lamotrigine and rifampicin (100 µM) demonstrated a moderated non-significant decrease in cell viability" should be re-written. If you have conducted statistics to show a trend then indicate the P value of the trend. A non significant difference means that there is no difference between the groups.

We thank the reviewer for this comment, this has been attended to as suggested

Page 17, Glucose (25 ,50 ,100 µM) – reposition the commas after the numbers to (25, 50,100 µM)

We thank the reviewer for this comment this has been fixed

One missing set of experiments is that the drug incubations should also have been completed on insulin treated myotubes. That would paint a more complete picture of the effects of these drug concentrations on myotube glucose handling as a whole.

We thank the reviewer for this insightful comment. The goal for this study was to broadly demonstrate how mitochondrial dysfunction through drugs affects insulin signalling pathway and glucose handling. Hence we aimed to understand how pre-exposure of cells to mitotoxic agents could lessen insulin effects. Our approach therefore aimed to pre-treat the cells with these mitotoxic drugs, followed by insulin treatment, after which measurements of parameters were conducted. In this way, we could be able to observe changes in insulin sensitivity post-exposure to mitotoxic agents. We believe through this approach, we have been able in part to prove that drug-induced mitochondrial dysfunction (using 6 different classes of drugs) does affect insulin sensitivity as evidenced by a decrease in insulin-stimulated glucose uptake, AKT, and GLUT 4 translocation. We believe the experiment proposed by a reviewer could be a significant stand-alone study, as it is a different approach, considering that the cells would be pre-treated with insulin, following by mitotoxic agents. As such, preliminary experiments will also be vital to establish time-points where one can observe the effects. We welcome the reviewer's comment, however from what we have outline above together with logistical issues associated studies of this nature, we believe this can be conducted as a follow-up study.

Reviewer #2: Through evaluating the intracellular mechanisms leading to the development of insulin resistance in the presence of various conventional pharmacological agents reported as potential mitochondrial toxicants in skeletal muscle cell line, the authors deciphered the effect of drug-induced mitochondrial dysfunction on insulin signalling cascade and glucose handling in C2C12 cell line in vitro. They used differentiated C2C12 preparations to be exposed to various concentrations of efavirenz, tenofovir, rifampicin, simvastatin, lamotrigine, and clarithromycin, separately, and then observed and measured the effect of drug-induced mitochondrial dysfunction on insulin signalling cascade. And they finally argued that the mitochondrial toxicants could potentially induce insulin resistance emanating from mitochondrial dysfunction. However, as indicated by the authors, the potential mitochondrial toxicants, efavirenz, tenofovir, rifampicin, simvastatin, lamotrigine, and clarithromycin have their own specific effects on insulin signalling cascade. Why is the same observation and measurement performed for all the mitochondrial toxicants?

We thank the reviewer for this insightful and interesting comment. Indeed, these agents could affect glucose metabolism through other affected pathways. However, what they have in common is disturbance in mitochondrial dysfunction. Although similar observations were reported, however, the extent on the effect was not the same, as some were more robust than others in affecting glucose handling and insulin signalling pathway. The same measurement was reported for the sake of uniformity and drawing comparison. The goal for now was to broadly demonstrate how mitochondrial dysfunction through commonly prescribed drugs affects insulin signalling pathway and glucose handling, therefore pose a risk of insulin resistance onset. We however believe this is a crucial point to take forward as we aim to further refine possible mechanisms utilized by these agents to alter glucose handling.
